# Dynamic contrast-enhanced computed tomography perfusion parameters of canine suspected brain tumors at baseline and during radiotherapy might be different depending on tumor location but not associated with survival

**DOI:** 10.3389/fvets.2023.1179762

**Published:** 2023-04-28

**Authors:** Jeremy R. Mortier, Thomas W. Maddox, Laura Blackwood, Matthew D. La Fontaine, Valeria Busoni

**Affiliations:** ^1^Small Animal Teaching Hospital, Institute of Infection, Veterinary and Ecological Sciences, University of Liverpool, Neston, United Kingdom; ^2^Diagnostic Imaging Section, Department of Clinical Sciences, Faculty of Veterinary Medicine, University of Liège, Liège, Belgium; ^3^The Netherlands Cancer Institute, Amsterdam, Netherlands

**Keywords:** perfusion parameters, dynamic contrast enhanced computed tomography, brain tumor, dogs, radiotherapy, response to treatment

## Abstract

**Introduction:**

Treatment of brain tumors in dogs can be associated with significant morbidity and reliable prognostic factors are lacking. Dynamic contrast-enhanced computed tomography (DCECT) can be used to assess tumor perfusion. The objectives of this study were to assess perfusion parameters and change in size of suspected brain tumors before and during radiotherapy (RT) depending on their location and find a potential correlation with survival.

**Methods:**

Seventeen client-owned dogs with suspected brain tumors were prospectively recruited. All dogs had a baseline DCECT to assess mass size, blood volume (BV), blood flow (BF), and transit time (TT). Twelve dogs had a repeat DCECT after 12 Gy of megavoltage RT. Survival times were calculated.

**Results:**

Intra-axial masses had lower BF (*p* = 0.005) and BV (*p* < 0.001) than extra-axial masses but not than pituitary masses. Pituitary masses had lower BF (*p* = 0.001) and BV (*p* = 0.004) than extra-axial masses. The volume of the mass was positively associated with TT (*p* = 0.001) but not with BF and BV. Intra-axial masses showed a more marked decrease in size than extra-axial and pituitary masses during RT (*p* = 0.022 for length, *p* = 0.05 for height). Extra-axial masses showed a greater decrease in BF (*p* = 0.011) and BV (*p* = 0.012) during RT than pituitary masses and intra-axial masses. Heavier dogs had a shorter survival time (*p* = 0.011). Perfusion parameters were not correlated with survival.

**Conclusion:**

DCECT perfusion parameters and change in size of brain masses during RT might be different based on the location of the mass.

## 1. Introduction

Brain tumors are a common cause of central neurological signs in dogs and associated with high morbidity (1). Radiotherapy (RT) (with or without surgical excision) or debulking is widely recognized as the treatment of choice, both to alleviate clinical signs and improve survival time (2–11). The survival advantage of surgery is controversial, and surgery is technically demanding and associated with significant morbidity (12–14). Survival times after RT are variable and affected by both treatment and tumor type, but a recent systematic review reported median survival times of 348 days, 226 days and 351 days for the extra-axial, intra-axial and pituitary masses, respectively (15). Radiotherapy is expensive, constraining for clients and can be associated with significant side effects due to early and late radiation toxicity (16, 17). It is therefore vital to find or develop good prognostic factors that would allow for optimal patient selection. Very few imaging-derived characteristics have been associated with the prognosis, although it seems that large masses (2, 7, 18), poorly defined and irregular margins, T2w heterogeneity, presence of drop metastasis (19), mass effect, and cyst-like lesions are negative prognostic factors (7). A decrease in size of the mass at 6 weeks after RT is also associated with a longer survival (20).

In human medicine, functional imaging is increasingly used in the diagnosis, treatment planning and prognostication of brain tumors, and includes magnetic resonance spectroscopy, diffusion-weighted magnetic resonance imaging (MRI), perfusion-weighted MRI and dynamic contrast-enhanced computed tomography (DCECT) (21, 22). Dynamic contrast-enhanced computed tomography provides quantitative information about tumor physiology that cannot be obtained using conventional imaging, in particular the blood volume (BV), blood flow (BF), and transit time (TT) of masses. In human medicine, it can be used to refine grading and prognostication of brain tumors, as well as assessing response to treatment (23, 24). To date, two studies in veterinary medicine described the use of DCECT in dogs with cerebral mass lesions, showing promising results in differentiating the type of masses and in assessing response to treatment (25, 26).

The aims of this study were (1) to compare DCECT-derived perfusion parameters among intra-axial, extra-axial and pituitary tumors, (2) to assess if pretreatment perfusion parameters are associated with survival, and (3) to describe early changes in perfusion parameters and size of the masses during RT in a subset of them. Our hypotheses were that extra-axial and pituitary tumors will have higher BF and BV than intra-axial tumors, that baseline perfusion parameters might be associated with survival time and that we can observe early changes in perfusion parameters and mass volume during RT.

## 2. Materials and methods

This is a prospective cross-sectional study. Ethical approval was granted by the Committee on Research Ethics at the Institute of Veterinary Science of the University of Liverpool (VREC560a).

### 2.1. Case selection

Client-owned dogs presented to the Small Animal Teaching Hospital (SATH) of the University of Liverpool for suspected brain tumors were prospectively enrolled from January 2017 to January 2020. Owner consent allowing for diagnostic tests including DCECT was obtained before inclusion into the study. To meet the inclusion criteria, dogs must have a brain mass on MRI and a presumptive diagnosis of brain tumor made by the attending board-certified radiologist and neurologist based on the imaging and clinical findings, but a final diagnosis of brain tumor made by histology was not necessary. Dogs must have undergone at least a baseline DCECT. Dogs who had already received RT, surgery or chemotherapy were excluded. Dogs receiving other non-chemotherapeutic medical treatments for their neurological signs (anti-inflammatory and anti-epileptic medication) were not excluded.

### 2.2. Clinical data

Treatment received at the time of DCECT, heart rate and systolic blood pressure during DCECT, treatment administered to treat the suspected brain tumor and survival time from the referral consultation were recorded. Treatment administered before the consultation was categorized as corticosteroids, non-steroidal anti-inflammatory drugs (NSAID) and other (paracetamol, anti-epileptic medication, trilostane) or none. Treatments received for the brain mass were categorized as RT or palliative (1 dog).

### 2.3. Dynamic contrast-enhanced computed tomography

All dogs were anesthetized. Premedication varied depending on the attending anesthetist, but most dogs received medetomidine (0.003 to 0.01 mg/kg) in association with butorphanol, lidocaine, buprenorphine or methadone. One dog received acepromazine (0.03 mg/kg) instead of medetomidine. Dogs were then induced using propofol or alfaxolone (to effect) and anesthesia was maintained using sevoflurane. Dynamic contrast-enhanced CT was performed using an 80-slice CT scan (Aquilion Prime 80, Canon Medical System) with dogs in sternal recumbency. Dogs for which owners elected for RT were positioned using a thermoplastic mask and a bite block as part as the RT planning (25).

Pre-contrast scans of the head were performed. Scanning parameters were 120 kV, variable mAs using Automatic Exposure Control, pitch factor 0.625, and images were reconstructed at 1 mm slice thickness using bone and soft tissue reconstruction algorithms. Dynamic contrast-enhanced CT planning was done using the pre-contrast soft tissue reconstruction in a soft tissue window (window width: 200 HU, window level 40 HU). A 4-cm length field of view was chosen to include the whole mass.

A 60-s continuous scan starting with intravenous injection of 2 mL/kg body weight of iodinated contrast medium (Ioversol 300 mg/mL iodine) using a power injector set at 3 mL/s injection rate (maximal allowable injection pressure set at 150 psi) and followed by a bolus flush of saline 1 mL/kg at the same injection rate. Scanning parameters were 80 kV, 200 mA, 0.75 s rotation time, 0.5 mm scan slice thickness, 1 s time interval and 2 mm reconstruction slice thickness. Images were reconstructed using a soft tissue reconstruction algorithm.

A post-contrast scan of the head was performed immediately after DCECT (90 s after intravenous injection of iodinated contrast medium), using the same scanning parameters as for the pre-contrast scan.

A second DCECT using the same anesthetic protocol (all under general anesthesia) and the same scanning technique was performed, after receiving 12 Gy of radiation.

### 2.4. Radiation therapy

Radiotherapy was administered using a linear accelerator (VitalBeam, Varian Medical Systems, Palo Alto, California). Definitive RT was administered with 12 fractions of 4 Gy on a Monday, Wednesday, Friday basis. All treatments were carried out at 6MV and were 3D planned by a European College of Veterinary Internal Medicine board-certified veterinary oncologist and radiation oncologist (LB). Planning was performed from CT images using Eclipse 15.1 (Varian Medical Systems, Palo Alto, California), with the intention to include at least 95% of the planning treatment volume in the 95 to 105% isodose, or 97% of the planning treatment volume in the 97 to 103% isodose. Gross tumor volume (GTV) and clinical target volume (CTV) were defined using CT and MRI. Gross tumor volume was contoured based on T1-weighted (or T1-weighted after IV administration of gadolinium) images. A margin of 1–2 mm of normal tissue, including questionable tissue, was added to the GTV to create the CTV. The CTV-margin was extended three-dimensionally by 3–5 mm to define the planning target volume (PTV). Organs at risk were contoured (tympanic bullae, brain, spinal cord, optic chiasma). Plans utilized 3 to 5 coplanar beams, with beam collimation using multileaf collimator beam modification and dynamic wedges where appropriate. Dogs were immobilized as described for the CT scans. Portal imaging was carried out at least twice during the treatment protocol to verify position.

### 2.5. Images and perfusion analysis

**Figure 1:**
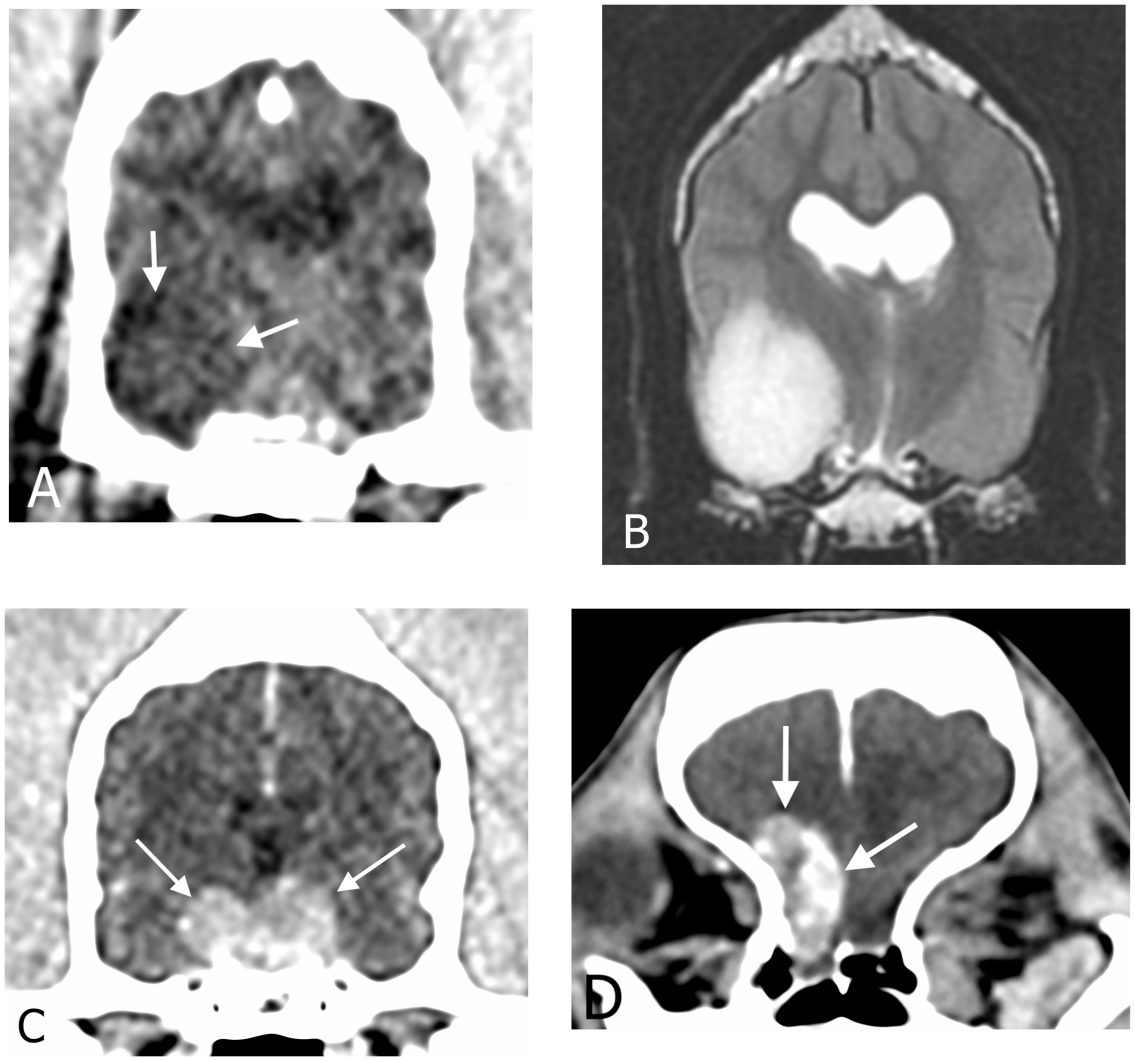
Transverse CT images in soft tissue reconstruction and brain window (WL 50 HU, WW 100 HU) showing **(A)** an intra-axial mass, **(C)** a pituitary mass and **(D)** an extra-axial mass. **(B)** Transverse T2-weighted magnetic resonance image of the same intra-axial mass as in **(A)**.

Conventional CT images of the head were reviewed by a European College of Veterinary Diagnostic Imaging board-certified veterinary radiologist (JM) blinded to the clinical data of the dogs, using a Macintosh workstation and an image viewer (OsirixMD, Pixmeo). Images were viewed using both a soft tissue window (window width: 200 HU, window level: 40 HU) and a brain window (window width: 100 HU and window level: 50 HU). Multiplanar reconstruction was performed for each dog. Length, width, and height with planes parallel and orthogonal to those of the head were measured and the volume of the mass (using the ellipsoid formula V = 4/3 × π × L × W × H) was calculated. In 2 dogs with an intra-axial lesion, the mass was not sufficiently visible on CT and T2w MRI sequences in transverse, sagittal and dorsal planes were used for initial morphological assessment. One of these dogs had a repeat DCECT but the mass could not be measured on the images. Masses were first classified as extra-axial, intra-axial or pituitary based on their location, relationship to the rest of the brain parenchyma and contrast-enhancement (Figure 1).

**Figure 2:**
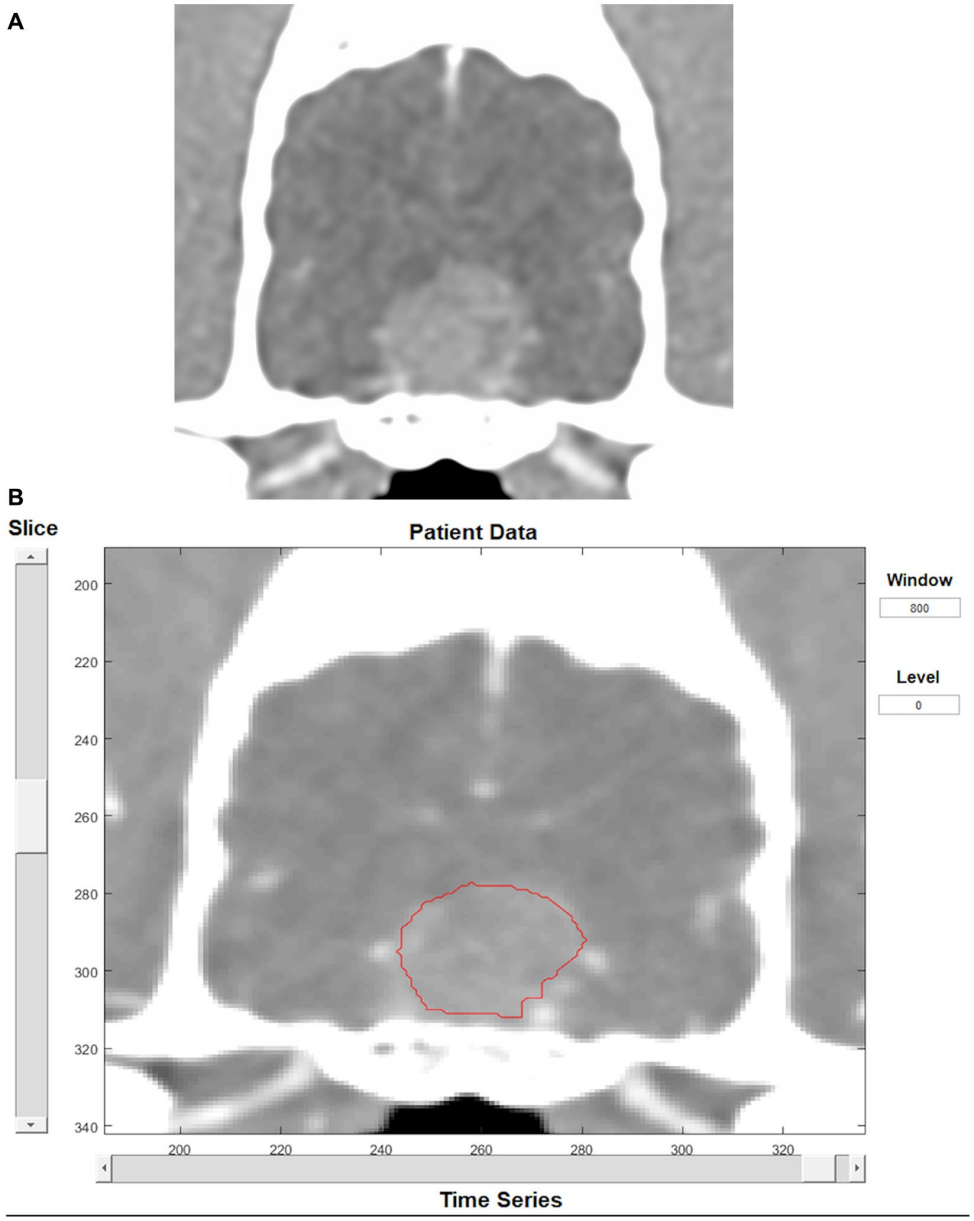
Transverse CT image in soft tissue reconstruction showing a pituitary mass **(A)**. Same image as displayed by the perfusion software, showing tumor contouring **(B)**.

Dynamic contrast-enhanced CT images were analyzed using an adiabatic approximation to the tissue homogeneity (ATH) model implemented with MATLAB^™^ (MathWorks, Massachusetts), designed as part of a previous study (27). An arterial input function was first contoured, and a time-attenuation curve was displayed to verify it had a shape consistent with arterial blood flow. The artery selected for the arterial input function was the lingual artery as it was the largest artery that was consistently included in the field of view and not surrounded by bone. To appropriately contour the artery without selecting peripheral lingual tissue the image was zoomed in and contoured on the arterial phase (veins not contrast-enhanced). Only the center of the artery was included when possible. The brain mass was then contoured manually slice by slice on every slice containing suspected tumoral tissue (Figure 2). Care was taken not to include bone or cerebral vessels within the contouring. Therefore, when present, the small part of the mass in contact with a cerebral vessel was excluded from contouring. For the two intra-axial masses not clearly visible on CT, side-by-side comparison of the MRI and CT images were performed to contour the mass as accurately as possible using the perfusion software. Perfusion parameters obtained from the analysis were blood flow (volume flow rate) and transit time (TT, time to traverse vasculature). Blood volume (BV) was then calculated using the following formula: BV = TT × BF. Perfusion analysis was performed by one trained operator (JM).

### 2.6. Statistical analysis

Statistical analyzes were performed using the statistical software programs SPSS 24.0 (SPSS Inc., Chicago, Illinois, United States) and R (R version 3.2.0, The R Foundation for Statistical Computing). Dependent (outcome) and independent variables were derived from signalment data, clinical data and CT examinations. Descriptive statistics were calculated for data as required; categorical variables were summarized as frequencies with 95% confidence intervals (95% CI) and continuous variables as medians with interquartile ranges (IQR). Categorical variables with multiple categories, categories containing small numbers, or both, were reviewed and groupings amalgamated if necessary. The distribution of continuous variables was assessed for deviation from normal both graphically and with the Kolmogorov–Smirnov test.

The three primary individual outcomes considered were BF, TT and BV, with associations between these continuous variables and the collected independent variables estimated with linear regression. All independent variables showing potential association with an outcome on univariable analysis (*p*-value <0.25) were considered for inclusion in the final multivariable model for that outcome. Variables showing evidence of correlation (correlation coefficient > 0.7), were examined and only the variable with the smallest *p*-value was selected for entry in the multivariable models developed. Final models were developed with a manual backwards stepwise methodology with retention of variables with *p*-values < 0.05. Additionally, for the 12 dogs that were re-scanned, linear regression was used to compare the change in tumor parameters (length, width, height, volume, BF, BV, TT) between pituitary, extra-and intra-axial tumors and to evaluate associations between tumor volume and perfusion parameters (BF, BV, TT).

Survival times were calculated from the time of the first consultation at the SATH until death; dogs alive at the time of data collection were considered as censored, as were dogs lost to follow-up (censored at the date of last known contact). Median survival times were calculated from Kaplan–Meier product limit (survival) analysis. Univariable comparison of survival times and associations between survival and independent variables were examined with the Log Rank test for categorical variables and Cox proportional hazard regression analysis for continuous variables. Multivariable regression models were constructed with the same approach as above, including all variables with univariable *p*-value <0.25 and a backward stepwise approach with retention of *p*-values <0.05.

## 3. Results

### 3.1. Clinical data

Seventeen dogs met the inclusion criteria. There were 4 neutered females, 3 entire males and 10 neutered males. Breeds included 4 cross breeds, 3 Staffordshire bull terriers, 2 Labrador retrievers and 1 dog of each breed including cocker spaniel, boxer, Cavalier King Charles spaniel, Dalmatian, Jack Russel terrier, pug, West Island white terrier and dachshund. Median age of the dogs was 9.9 (6.6–14.3) years; median weight was 19.9 (6.6–59.6) kg.

There were 7 pituitary masses, 6 extra-axial masses and 4 intra-axial masses. Among the extra-axial masses, 4 were localized against the basicranium, 1 at the cerebellar convexity and 1 at the cerebral convexity. Among the intra-axial masses, 2 were within the temporal cortex, 1 within the cerebellum and 1 within the thalamus.

Nine dogs received corticosteroids (4 extra-axial, 2 intra-axial, 2 pituitary), 1 dog received NSAID (1 pituitary), 2 dogs received “other” treatments (one paracetamol and gabapentin; one trilostane) and 5 dogs had no treatment at the time of presentation (2 pituitary, 2 extra-axial, 1 intra-axial).

Twelve/17 dogs had repeat DCECT. There were 5 pituitary masses, 5 extra-axial masses and 2 intra-axial masses. Five dogs could not have repeat DCECT for logistical reasons. One did not received RT, DCECT failed in 1 dog and the remaining dogs were not considered stable enough to undergo the procedure.

At repeat DCECT, 10/12 dogs were receiving corticosteroids, 1 dog was on phenobarbital (1 intra-axial) only and 1 dog was not receiving any medical treatment (1 extra-axial). Most dogs were receiving analgesics in addition to the aforementioned treatment.

Sixteen/17 dogs had RT, and 1 dog did not receive any treatment for the suspected brain tumor. Median time between first DCECT and start of RT was 6 days (3–31).

Twelve/17 dogs had a record of their heart rate during the baseline DCECT, at the time of injection of contrast medium. All were considered appropriate by the attending anesthetist and ranged from 40 to 110 bpm (median 68 bpm). Similarly, systolic blood pressure was obtained for 12 dogs and ranged from 90 to 130 mmHg (median 115 mmHg).

### 3.2. Baseline DCECT

Median mass length, width, height, and volume were 1.4 cm (0.8–3.2), 1.2 cm (0.6–2.2), 1.2 cm (0.4–2.1) and 8.2 cm^3^ (1.4–29.6), respectively. Median volume was 16 cm^3^ (1.8–25.5) for pituitary masses, 9.4 cm^3^ (1.5–15.8) for extra-axial masses, and 8 cm^3^ (1.4–29.6) for intra-axial masses.

Median BV was 3.6 ml/100 g (0.7–11), median BF was 33.4 ml/100 g/min (2.8–116.1) and median TT was 7.2 s (4.1–15.7). Results for the different locations of masses are shown in Table 1. Intra-axial masses had a significantly lower BF (*p* = 0.005) and BV (*p* < 0.001) than extra-axial masses but not than pituitary masses. Similarly, pituitary masses had significantly lower BF (*p* = 0.001) and BV (*p* = 0.004) than extra-axial masses. The volume of the mass was positively associated with TT (*p* = 0.001) but not with BF and BV. There was no statistical association between any other imaging characteristic of the masses and the perfusion parameters.

**Table 1:** Median baseline perfusion parameters of brain tumors in dogs depending on their location.

Mass location	Blood volume (min; max) (mL/100 g)	Blood flow (min; max) (mL/100 g/min)	Transit time (min; max) (s)
Intra-axial	1.7 (0.7; 2.5)	22.4 (2.8; 33.4)	4.8 (4.1; 5.1)
Extra-axial	7.1* (3.8; 11)	54* (34.4; 116.1)	6.9 (5.2; 9.5)
Pituitary	3.6 (1.8; 4.3)	22.6 (9.6; 50)	8.8 (4.6; 12.4)

*p < 0.05.

### 3.3. Repeat DCECT

Median change in volume, length, height, width for each type of mass is shown in Table 2. There were statistically significant differences in the change of length and height but not width and volume between extra-axial, intra-axial and pituitary masses during the course of RT. More specifically, intra-axial tumors showed a more marked decrease in size than extra-axial and pituitary tumors during RT (*p* = 0.022 for the length and *p* = 0.05 for the height).

**Table 2:** Median change in the size of brain masses after 12Gy of radiation therapy depending on their location.

Mass location	Change in volume (min; max) (cm^3^)	Change in length (min; max) (cm)	Change in height (min; max) (cm)	Change in width (min; max) (cm)
Extra-axial	−1.6 (−1.7; 0.9)	0 (−0.2; 0)	0 (0; 0.6)	0 (−0.8; 0)
Intra-axial	−8.2 (−1; −0.1)	−0.3* (−1.3; −0.3)	−0.3* (−1.5; −0.3)	−0.4 (−1; −0.1)
Pituitary	−0.1 (−5; 0.1)	−0.1 (−0.3; 0.1)	0 (−0.3; 0)	−0.1 (−0.1; 0.1)

*p < 0.05.

The median change in perfusion parameters for each type of mass are presented in Table 3. There were statistically significant differences in the change of BF and BV but not TT between extra-axial, intra-axial and pituitary masses during the course of RT. More specifically, extra-axial tumors showed a more marked decrease in BF (*p* = 0.011) and BV (*p* = 0.012) than pituitary tumors and intra-axial tumors.

**Table 3:** Median change in perfusion parameters of brain masses after 12Gy of radiation therapy depending on their location.

Mass location	Change in blood volume (min; max) (mL/100 g)	Change in blood flow (min; max) (mL/100 g/min)	Change in transit time (min; max) (s)
Extra-axial	−2.4* (−3.1; −1.4)	−24.7* (−54.6; −11.4)	1,5 (−0.42; 2.19)
Intra-axial	0.3 (0.1; 2.1)	18.2 (−2.5; 50.4)	−1.1 (−12.4; 0.6)
Pituitary	0.6 (−2.1; 1.6)	6.4 (3.7; 38.3)	−1.1 (−7.3; 1.6)

*p < 0.05.

There was no statistically significant association between tumor volume change and change in BF, BV or TT.

### 3.4. Survival analysis

At the end of the study, 11 dogs were deceased, all due to progression of their intracranial mass, and six were either still alive or lost to follow-up.

The median survival time was 186 days (95%CI 0–496) for all dogs. The only dog who did not receive RT had a survival time of 175 days. There was no statistical difference in survival time between intra-axial, extra-axial and pituitary masses.

On multivariate analysis, only the weight of the dog was associated with survival (*p* = 0.011). More specifically, heavier dogs had a shorter survival time. Perfusion parameters were not significantly associated with survival in the final statistical model.

## 4. Discussion

The perfusion parameters of presumed brain tumors were significantly different depending on their location. Extra-axial masses had higher BF and BV than intra-axial and pituitary masses. In the early course of RT (after administration of 12 Gy), intra-axial masses showed a greater decrease in size than masses of the other groups, and extra-axial masses had a more marked decrease in BF and BV than masses of the other groups.

A study on 16 dogs with various types of brain masses (including 3 meningiomas and 7 gliomas but no pituitary masses) showed that meningiomas had the highest BV among all masses, although their results were not statistically significant (25). The authors also found that the permeability surface area ratio (PS) was higher in meningioma than in other brain masses, but they did not calculate BF. Although the DCECT scanning protocol was similar to the one used in the present study, the method of perfusion analysis was not described in detail and may have been different, making comparisons of the results difficult. Higher BF and BV in extra-axial masses compared to intra-axial masses is consistent with expectations, as the former are located outside the blood–brain barrier, therefore tend to be hypervascular and have permeable capillaries (29). Although perfusion imaging is not commonly used to differentiate intra-axial from extra-axial masses in human medicine, their findings were comparable to those of this study (29, 30). Pituitary masses are also considered extra-axial and frequently have strong contrast enhancement (31), suggesting hypervascularity, therefore high BV and BF would have been expected. In the present study, their perfusion values were high but not significantly different from those of intra-axial masses. This may be due to the small number of cases. Perfusion imaging is rarely performed on pituitary adenomas in human beings, therefore comparison with human studies is not possible (32).

A more recent study investigated the change in volume and perfusion parameters of brain tumors (confirmed and suspected) before and approximately 3 months and 6 months after RT (26). They found a significant decrease in tumor volume, BF, BV, and PS after RT, maintained or further decreased at second recheck. These results combined with those of this study suggest that the decrease in DCECT perfusion parameters occurs early during RT and is at least maintained if not progressing further during the first 6 months after treatment.

The fact that intra-axial masses showed a greater decrease in size than extra-axial and pituitary masses was interesting and unexpected. However, this result should be interpreted with caution given the small number of dogs in this group and the technical difficulty in accurately measuring intra-axial masses on CT. Imaging follow-up during or after treatment of brain tumor is rare and unstandardized in veterinary research and studies assessing the association between change in volume, type of tumor and survival are scarce. It seems that a reduction in size of gliomas 6 weeks after RT is associated with longer survival (20). These results were not consistent with a previous study on various brain tumors that suggested that pituitary masses could be associated with a greater decrease in volume than other tumor types (glioma, meningioma and trigeminal nerve sheath tumors) 3 months after RT (26). It is therefore possible that gliomas have a greater initial size reduction, but that pituitary tumors have a greater delayed response. Direct tumor toxicity of RT through DNA damage is related to cell multiplication, and more aggressive tumors with fast-multiplying cells generally respond better to RT (33). On the other hand, indirect RT toxicity through its action on tumor microenvironment (such as vascularization and local immune system) could explain a delayed response (34). It could be hypothesized that the intra-axial masses in this study could be more aggressive than the extra-axial and pituitary masses, therefore have a better early response to treatment but also a faster tumor repopulation.

Extra-axial masses had a greater decrease in BF and BV during RT than the other tumor groups. This could simply be explained by the fact that extra-axial masses were more perfused at baseline, therefore the toxicity of RT on vascularity could have been more pronounced in this group. However, the response of the tumor microenvironment to RT is a complex and still poorly understood topic, and oversimplified assumptions should be avoided. Relationships between baseline perfusion parameters, their variation after treatment and prognosis have been investigated in people with high-grade gliomas. High-grade gliomas tend to have higher baseline perfusion parameters than low-grade gliomas, while lower post-treatment perfusion parameters were associated with a better prognosis (23, 24, 35–39). In the current study, the fact that the baseline and follow-up perfusion parameters were not associated with the change in size of the mass and the survival of the dogs could be due to the small population of dogs, the fact that all brain masses were considered as a single group when assessing the statistical relationship between these parameters, but also because of the variability of clinical signs associated with brain masses, leading to humane euthanasia regardless of response to treatment or aggressivity of the tumor. A study in a much larger population would be needed to further assess the potential prognostic role of DCECT perfusion parameters in brain tumors.

Heavier dogs had shorter survival in this study, regardless of tumor location. There is no clear reason to explain this finding, but it could simply be that heavier dogs with severe neurological clinical signs would be less manageable by their owners, therefore euthanized earlier in the course of the disease. Alternately, it could also represent a bias due to the small number of dogs included.

This study has many limitations. The main one is the small number of dogs, limiting the statistical power. A second limitation is the absence of confirmation and characterization of the masses. Using only the imaging characteristics, it is often impossible to differentiate between meningioma and histiocytic sarcoma, between pituitary adenoma and carcinoma, and between types and grades of gliomas (31, 40, 41). Unfortunately, brain biopsy requires specific equipment and experience and is not routinely performed in veterinary medicine, and a post-mortem examination is often declined by the owners. The accuracy of MRI has been reported to be as high as 100% in predicting the presence of a brain tumor (40), and 70 to 96% accurate in predicting the correct tumor type (42, 43). However, several studies in the veterinary literature report that other types of lesions such as cerebral vascular accidents can be misdiagnosed as gliomas on MRI (44, 45). Therefore, the diagnosis of brain tumor remains presumptive in this study.

Some dogs received ant-inflammatory drugs before first or repeat DCECT, or before both; other did not. The effects of anti-inflammatory drugs on DCECT perfusion parameters have not been studied, yet their anti-COX-2 activity have an anti-angiogenic action and could be responsible for changes in the perfusion parameters (46).

The technique of DCECT images acquisition and analysis used in this study also has some inherent limitations. The DCECT protocol used meets the human recommendations except for the injection rates of contrast medium and saline flush. Indeed, due to catheter size limitations leading to overpressure during injection, a 5 ml/s injection rate was not feasible. However, the smaller size of dogs compared to human beings likely balances out this limitation. Finally, intra and inter-observer variability have not been calculated in this study but would have been interesting, especially since it represents the highest contributor to overall variability in DCECT (47). A study found a coefficient of variation within patient ranging from 22 to 30% due to variability of the arterial input function area under the curve and variability in the tumor area under the curve in dogs with nasal tumors, using the same perfusion analysis software as the one used in this study (27). On the other hand, patients’ blood pressure was not found to have a significant impact on the perfusion analysis.

Contouring of masses close to the calvarium obliged to exclude a sliver of tissue to avoid including bone in the perfusion analysis. Another important technical limitation is the fact that in two dogs with an intra-axial mass, contouring was performed where the observer subjectively thought the mass was based on MRI studies, and therefore might have been inaccurate.

In conclusion, this study found an association between the location of intracranial masses and the perfusion parameters at baseline (extra-axial masses have higher BF and BV than intra-axial and pituitary masses) and during RT (with extra-axial tumors showing a higher decrease in BF and BV than pituitary and intra-axial tumors), and between the location of the mass and the size reduction during RT (with intra-axial tumors showing a higher decrease in size than extra-axial and pituitary tumors). Functional imaging, including DCECT, is of increasing availability in veterinary medicine yet still poorly studied. Further research in this field might be extremely valuable, with potential to help refine the diagnosis and prognosis, guide the choice of treatment, assess the treatment response, and detect recurrence in dogs with brain tumors.

## Data availability statement

The raw data supporting the conclusions of this article will be made available by the authors, without undue reservation.

## Ethics statement

The animal study was reviewed and approved by Research Ethics at the Institute of Veterinary Science of the University of Liverpool (VREC560a). Written informed consent was obtained from the owners for the participation of their animals in this study.

## Author contributions

JM, TM, VB, LB, and ML: conception and design. JM and LB: acquisition of data. JM and TM: analysis and interpretation of data. JM, LB, and TM: drafting the article. JM, TM, VB, LB, and ML: revising article for intellectual content, final approval of the completed article, and agreement to be accountable for all aspects of the work in ensuring that questions related to the accuracy or integrity of any part of the work are appropriately investigated and resolved. All authors contributed to the article and approved the submitted version.

## Funding

The authors received a research grant from the University of Liège for this work (FSR 2018).

## Conflict of interest

The authors declare that the research was conducted in the absence of any commercial or financial relationships that could be construed as a potential conflict of interest.

## Publisher’s note

All claims expressed in this article are solely those of the authors and do not necessarily represent those of their affiliated organizations, or those of the publisher, the editors and the reviewers. Any product that may be evaluated in this article, or claim that may be made by its manufacturer, is not guaranteed or endorsed by the publisher.

## Abbreviations

BF: blood flow
BV: blood volume/fractional vascular volume
CT: computed tomography
CTV: clinical target volume
DCECT: dynamic contrast-enhanced computed tomography
GTV: gross tumor volume
MRI: magnetic resonance imaging
PTV: planning target volume
TT: transit time
NSAID: non-steroidal anti-inflammatory drugs
RT: radiotherapy
